# Developing and Validating an Age-Independent Equation Using Multi-Frequency Bioelectrical Impedance Analysis for Estimation of Appendicular Skeletal Muscle Mass and Establishing a Cutoff for Sarcopenia

**DOI:** 10.3390/ijerph14070809

**Published:** 2017-07-19

**Authors:** Yosuke Yamada, Miyuki Nishizawa, Tomoka Uchiyama, Yasuhiro Kasahara, Mikio Shindo, Motohiko Miyachi, Shigeho Tanaka

**Affiliations:** 1 Department of Nutrition and Metabolism, National Institute of Health and Nutrition, National Institutes of Biomedical Innovation, Health and Nutrition, 1-23-1 Toyama, Shinjuku-ku, Tokyo 162-8636, Japan; tanakas@nibiohn.go.jp; 2 TANITA Body Weight Scientific Institute, TANITA Corporation, 1-14-2 Maeno, Itabashi-ku, Tokyo 174-8630, Japan; miyuki.kodama.p@tanita.co.jp (M.N.); tomoka.uchiyama@tanita.co.jp (T.U.); kasayasu@tanita.co.jp (Y.K.); mikio.shindo@tanita.co.jp (M.S.); 3 Department of Physical Activity Research, National Institute of Health and Nutrition, National Institutes of Biomedical Innovation, Health and Nutrition, 1-23-1 Toyama, Shinjuku-ku, Tokyo 162-8636, Japan; miyachi@nibiohn.go.jp

**Keywords:** age-related skeletal muscle loss, sarcopenia, malnutrition risk assessment, DXA, multi-frequency BIA, aging

## Abstract

*Background:* Appendicular skeletal muscle (or lean) mass (ALM) estimated using dual-energy X-ray absorptiometry (DXA) is considered to be a preferred method for sarcopenia studies. However, DXA is expensive, has limited portability, and requires radiation exposure. Bioelectrical impedance analysis (BIA) is inexpensive, easy to use, and portable; thus BIA might be useful in sarcopenia investigations. However, a large variety of models have been commercially supplied by different companies, and for most consumer products, the equations estimating ALM are not disclosed. It is therefore difficult to use these equations for research purposes. In particular, the BIA equation is often age-dependent, which leads to fundamental difficulty in examining age-related ALM loss. The aims of the current study were as follows: (1) to develop and validate an equation to estimate ALM using multi-frequency BIA (MF-BIA) based on theoretical models, and (2) to establish sarcopenia cutoff values using the equation for the Japanese population. *Methods:* We measured height (Ht), weight, and ALM obtained using DXA and a standing-posture 8-electrode MF-BIA (5, 50, 250 kHz) in 756 Japanese individuals aged 18 to 86-years-old (222 men and 301 women as developing equation group and 97 men and 136 women as a cross validation group). The traditional impedance index (Ht^2^/Z_50_) and impedance ratio of high and low frequency (Z_250_/Z_5_) of hand to foot values were calculated. Multiple regression analyses were conducted with ALM as dependent variable in men and women separately. *Results:* We created the following equations: ALM = (0.6947 × (Ht^2^/Z_50_)) + (−55.24 × (Z_250_/Z_5_)) + (−10,940 × (1/Z_50_)) + 51.33 for men, and ALM = (0.6144 × (Ht^2^/Z_50_)) + (−36.61 × (Z_250_/Z_5_)) + (−9332 × (1/Z_50_)) + 37.91 for women. Additionally, we conducted measurements in 1624 men and 1368 women aged 18 to 40 years to establish sarcopenia cutoff values in the Japanese population. The mean values minus 2 standard deviations of the skeletal muscle mass index (ALM/Ht^2^) in these participants were 6.8 and 5.7 kg/m^2^ in men and women, respectively. *Conclusion:* The current study established and validated a theoretical and age-independent equation using MF-BIA to estimate ALM and provided reasonable sarcopenia cutoff values.

## 1. Introduction

Life span has dramatically increased worldwide [1], and it is projected that the population of older adults aged 65 and over will be approximately 40% in 2055 in Japan [2]. The incidence and prevalence of many diseases, such as metabolic dysfunction, mobility disorders, and frailty increase with ageing [3], and thus, aging of the population leads to increased medical and long-term care costs. Skeletal muscle is the largest organ in the body, and maintenance of its quality and mass is essential for the prevention of age-related decline in metabolic function and physical frailty [4,5].

Recently, several international working groups established diagnosis or screening cutoff values for sarcopenia, i.e., age-related muscle mass loss and loss of muscle strength and/or physical function [6,7,8,9]. The prevention or improvement of sarcopenia is the target of nutrition and/or exercise interventions in aging. In the diagnosis and screening of sarcopenia, the primary measurement used is skeletal muscle mass (SMM). According to the European Working Group on Sarcopenia in Older People (EWGSOP), X-ray computed tomography (CT) and magnetic resonance imaging (MRI) are gold standards for estimating SMM, and appendicular lean mass (ALM) measured by dual-energy X-ray absorptiometry (DXA) is considered to be an alternative preferred method and index for research and clinical use [7]. However, these methods are expensive, have limited portability, and with the exception of MRI, require radiation exposure. They are thus limited to use in routine health examinations.

Bioelectrical impedance analysis (BIA) is inexpensive, easy to use, portable, and requires no radiation exposure [10,11,12,13,14,15,16,17,18,19,20,21,22,23,24]. Thus, BIA may be useful as a portable alternative to DXA [25,26,27,28,29]. However, a large variety of models have been commercially supplied by different companies, and for most consumer products, the equations used to estimate ALM or SMM are not disclosed, making them difficult to use for research purposes [25]. In particular, the BIA equations are often age-dependent [30], which leads to fundamental difficulty in diagnosing age-related ALM loss. The aims of the current study were as follows: (1) to develop and validate an age-independent equation for estimation of ALM using multi-frequency BIA (MF-BIA) based on theoretical models, and (2) to establish cutoff values for sarcopenia using the equation for the Japanese population.

Skeletal muscle holds a large volume of water [31] and the water in skeletal muscle is distributed to both extra- and intracellular compartments (ECW and ICW, respectively) partitioned by muscle cell membranes [32]. Previous studies indicated that a relative expansion of ECW against ICW is observed in skeletal muscles with aging [32,33,34]. In addition, increased edema would influence the estimation of SMM by BIA [35,36,37]. Our hypothesis is that if we consider the effect of water distribution and the possibility of edema in the estimation model, theoretically, we can develop an age-independent equation based on MF-BIA for estimation of ALM.

## 2. Materials and Methods

### 2.1. Participants

For developing the equation and examining its validity, a total of 756 Japanese individuals who were undergoing a company health examination aged 18 to 86 years (222 men and 301 women as the developing equation group and 97 men and 136 women as the cross validation group) were enrolled in this study. The study protocol was approved by the ethics committee of the company, TANITA Co. IRB, and the approval numbers were #004, 005, 010 and 012. All participants gave written informed consent after reviewing the purpose, methods, and significance of the study. We measured height (Ht), weight, DXA, and MF-BIA. Additionally, we measured MF-BIA of 1624 men and 1368 women aged 18 to 40 years to establish a sarcopenia cutoff value for the Japanese population. Inclusion criteria were as follows: (1) reported ability to walk more than 10 m with or without a cane; (2) ability to provide informed consent with no indication of dementia; (3) no history of joint arthroplasty or current use of an artificial pacemaker.

### 2.2. Multi-Frequency Bioelectrical Impedance Analysis

A standing-posture 8-electrode MF-BIA (MC-780A-N, TANITA, Tokyo, Japan) was used to measure bioelectrical impedance (Z) at 5, 50 and 250 kHz frequency (Z_5_, Z_50_, and Z_250_, respectively). Routine quality assurance procedures were conducted using a custom-made impedance tester and no instrument drift or shift was detected during the measurement period. Participants were evaluated in their underwear, in a standing position, and were asked to stand barefoot on toe-and heel electrodes and to hold the handgrips with arms hanging down a few centimeters from the hip. The eight-electrode method enables segmental impedance measurement. The electrical current is ≤90 μA. Minimum weight graduation was 0.1 kg. Participants were instructed to refrain from vigorous exercise and consuming alcohol for the 24-h period before the experiment, to finish the last meal at least 2.5-h before the measurement, and to empty their bladder before the measurement. Measurements were conducted between 15:00 to 17:30, and room temperature was adjusted to maintain a thermoneutral environment.

Most BIA devices and equations use an impedance index (Ht^2^/Z_50_) or resistance index of 50 kHz (Ht^2^/R_50_) calculated by Ht and Z_50_ or resistance at 50 kHz (R_50_) as a predicting variable for AMM or ALM [17,30,38]. In the human body, reactance is less than 10% of resistance, and correlation between impedance and resistance are >0.99; thus Ht^2^/Z_50_ and Ht^2^/R_50_ are interchangeable. We chose to use Ht^2^/Z_50_ as a candidate independent variable to estimate ALM in this study. One of the most well-known equations for estimating muscle mass by BIA was developed by Janssen et al. using MRI as follows: SMM (kg) = ((Ht^2^/R_50_ × 0.401) + (sex × 3.825) + (age × −0.071)) + 5.102 [30], and many BIA equations are age-dependent. Yamada et al. indicated that expansion of ECW relative to ICW or total body water (TBW) is observed with aging, and may mask actual age-related decrease of muscle cell mass [32,34,39,40,41]. The Z at low-frequency (≤50 kHz, for example, Z_5_ or Z_50_) currents mainly reflects ECW. In contrast, the Z at high-frequency (≥250 kHz) reflects TBW. Thus, the impedance ratio of Z_250_ against Z_5_ (Z_250_/Z_5_) is an index of ECW/TBW, and we chose to use Z_250_/Z_5_ as another candidate independent variable to estimate ALM. In addition, the possibility of edema affects ALM estimation, i.e., if the person has edema, particularly peripheral edema, the BIA overestimates actual ALM. The index of 1/Z_5_ or 1/Z_50_ could potentially be applied as an adjusting variable for this situation. Thus, we selected 1/Z_50_ as an additional candidate independent variable to estimate ALM. We developed estimating equations for men and women separately, because body composition and fat and muscle distribution are quite different between men and women.

### 2.3. Dual-Energy X-ray Absorptiometry

A Lunar DPX-L (GE Healthcare, Madison, WI, USA) densitometer was used and analyses were performed using the software version of 1.35 (Lunar DPX-L ver1.35, GE Healthcare Lunar, Madison, WI, USA) to obtain ALM. Routine densitometry quality assurance procedures were conducted using a standard phantom once per week and calibrated before the test, and no instrument drift or shift was detected during the measurement period.

### 2.4. Statistical Analysis

Results are presented as means ± standard deviations (SD) with maximum and minimum values. As the physical characteristics were different between sexes, the statistical analyses were applied to men and women separately. To examine the association between age and other variables, quadratic regression analyses were conducted with ALM, Ht^2^/Z_50_, and Z_250_/Z_5_ against age, and determination coefficients were obtained. To examine the effect of age on BIA estimation, we investigated the association between age and the residual of the ALM obtained by DXA and the ALM estimated by one of the previous BIA equations for Japanese older adults aged 65 years and over using TANITA BIA according to the following Equations [38]:

Men: ALM = (0.197 × (Ht^2^/Z_50_)) + (0.179 × Weight) − 0.019 (1)

Women: ALM = (0.221 × (Ht^2^/Z_50_)) + (0.117 × Weight) + 0.881 (2)

Multiple linear regression analyses were conducted for the equation developing groups using Ht^2^/Z_50_, Z_250_/Z_5_, and 1/Z_50_ as independent variables and ALM as the dependent variable, where entry probability of F was 0.05 and removal was 0.10. To validate the newly developed equation, the association between the ALM estimated with the newly developed MF-BIA equation and the ALM obtained by DXA was examined in the validation groups and pair t-test was conducted between measured and estimated ALM. To establish sarcopenia cutoff values based on Baumgartner et al. [42], we calculated the mean minus 2SD in 1624 men and 1368 women aged 18 to 40 years using the equation. All analyses were performed using SPSS software (Version 22.0 for Windows, IBM Corp., Armonk, NY, USA). For all analyses, *p* < 0.05 was used to indicate statistical significance.

## 3. Results

The physical characteristics and BIA variables of study participants are presented in Table 1. Height, weight, body mass index (BMI), ALM, Z_5_, Z_50_, Z_250_, Ht^2^/Z_50_, Z_250_/Z_5_ and 1/Z_50_ were significantly different between men and women.

**Figure 1:**
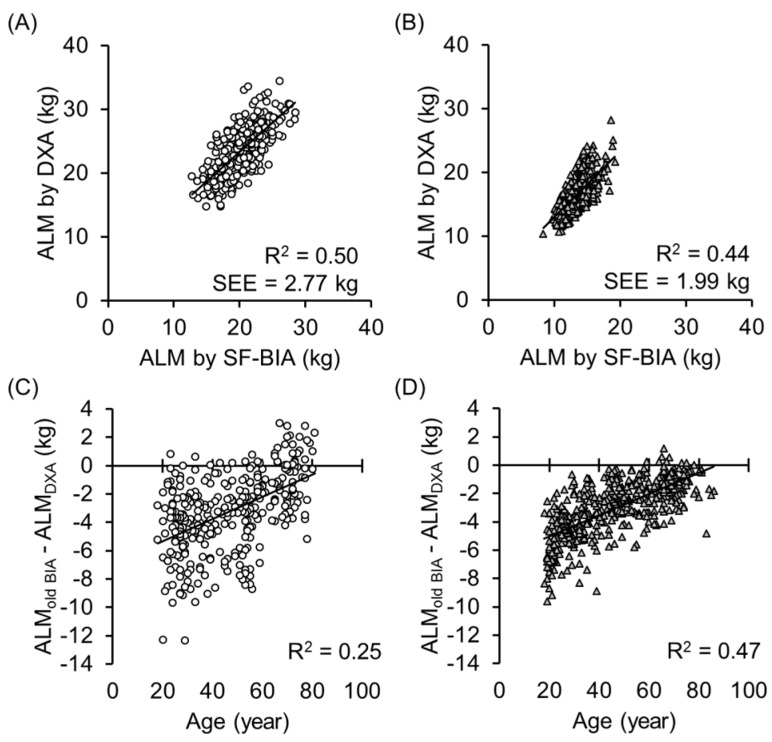
A and B shows the relationship between the appendicular lean mass (ALM) estimated by one of the previous bioelectrical impedance analysis equation for a standing-posture 8-electrode TANITA MF-BIA and the ALM obtained dual X-ray absorptiometry (DXA) in 319 men (**A**) and 437 women; (**B**) The equation was established and validated for Japanese older adults aged 65y and over as a previous equation by [38]. The determinant coefficient were just moderate (R^2^ = 0.50 and 0.44, men and women, respectively) because of the wide range of the age in the current participants (18 to 86 years). (**C**,**D**) shows the relationship between age and the residual of the ALM obtained DXA and the ALM estimated by the previous BIA equation. The residual was significantly correlated with age, which indicated that the previous BIA equation underestimated ALM in young adults. The previous equation cannot be used to examine age-related ALM loss.

Figure 1A,B shows the association between the ALM estimated by the previous BIA equation (see methods section) and the ALM obtained by DXA. The determinant coefficients were just moderate (R^2^ = 0.49 and 0.44 for men and women, respectively). Figure 1C,D shows the correlation between age and the residual of the ALM obtained by DXA and the ALM estimated by the previous BIA equation. The residual significantly correlated with age, and the previous BIA equation underestimated ALM in young adults. Thus, the previous equation cannot be used to examine age-related ALM loss.

**Figure 2:**
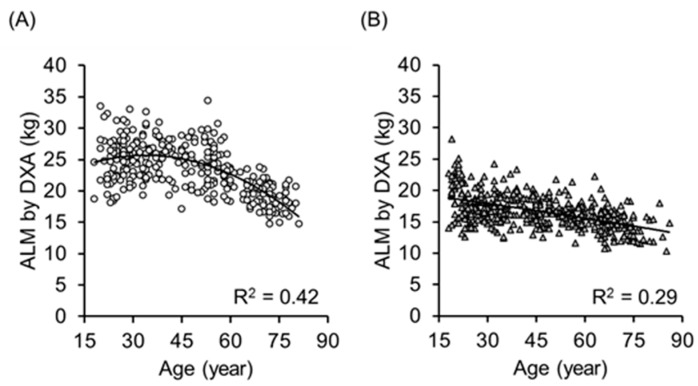
Relationship between age and the appendicular lean mass (ALM) obtained by DXA in 319 men (**A**) and 437 women (**B**) aged 18 to 86 years old. The determinant coefficients of quadratic regression analysis were R^2^ = 0.42 and 0.29, respectively.

**Figure 3:**
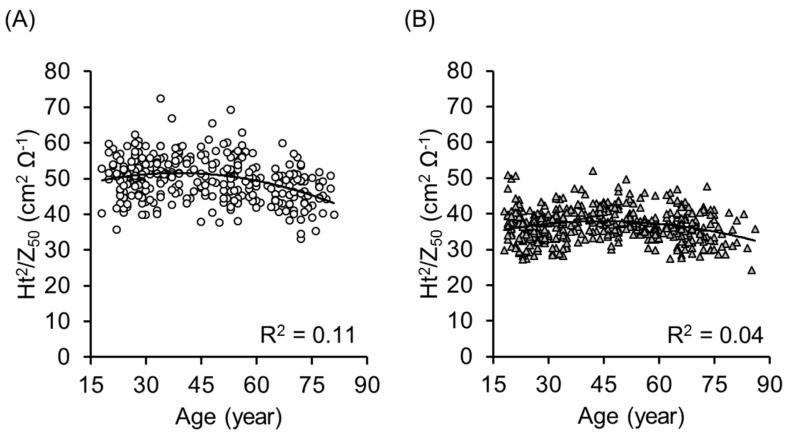
Relationship between age and the impedance index (height squired divided by impedance at 50 kHz: Ht^2^/Z_50_) obtained by bioelectrical impedance analysis in 319 men (**A**) and 437 women (**B**) aged 18 to 86 years old. The determinant coefficients of quadratic regression analysis were R^2^ = 0.11 and 0.04, respectively, which is significantly lower than that of the age-ALM relationship in DXA.

Figure 2 shows the association between age and the ALM obtained by DXA in 319 men (A) and 437 women (B). The determinant coefficients of quadratic regression analysis were R^2^ = 0.42 and 0.29 in men and women, respectively. In contrast, as Figure 3 shows, the determinant coefficients of quadratic regression analysis between age and Ht^2^/Z_50_ obtained by BIA were R^2^ = 0.11 and 0.04 in men and women, respectively.

**Figure 4:**
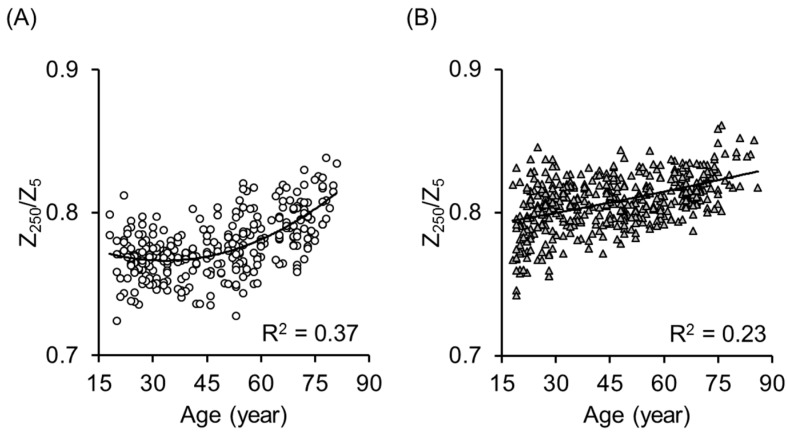
Relationship between age and the impedance ratio of high and low frequency (impedance at 250 kHz divided by impedance at 5 kHz: Z_250_/Z_5_) obtained by multi-frequency bioelectrical impedance analysis (MF-BIA) in 319 men (**A**) and 437 women (**B**) aged 18 to 86 years old. The determinant coefficients of quadratic regression analysis were R^2^ = 0.37 and 0.23, respectively. The Z_250_/Z_5_ is the index of the ratio between extra- and intra-cellular water compartments in the body.

Figure 4 shows the quadratic association between age and the index (Z_250_/Z_5_) of the ratio between extra- to intra-cellular water compartments obtained by MF-BIA in men (A) and women (B). The determinant coefficients of quadratic regression analysis were R^2^ = 0.37 and 0.23, respectively.

Table 2 shows the results of multivariate analysis of the linear model with ALM as a dependent model in the developing equation groups for men and women, respectively. In model 1, Ht^2^/Z_50_ was a significant independent variable to estimate ALM, but the determinant coefficient was moderate and the standard error of estimation (SEE) was relatively large (2.27 and 1.88 kg in men and women, respectively). In contrast, in the final model (model 3), all impedance variables became significant independent variables to estimate ALM, the determinant coefficients became significantly greater than model 1, and SEE became lower than models 1 and 2 (1.46 and 1.22 kg in men and women, respectively). The variance inflation factor (VIF), an index to detect multicollinearity, was less than 5, so that the final model is acceptable to use. The negative coefficients of 1/Z_50_ indicated that 1/Z_50_ did work as a suppressor variable in the model.

The final equations were following:

Men: ALM = (0.6947 × (Ht^2^/Z_50_)) + (−55.24 × (Z_250_/Z_5_)) + (−10940 × (1/Z_50_)) + 51.33 (3)

Women: ALM = (0.6144 × (Ht^2^/Z_50_)) + (−36.61 × (Z_250_/Z_5_)) + (−9332 × (1/Z_50_)) + 37.91 (4)

**Figure 5:**
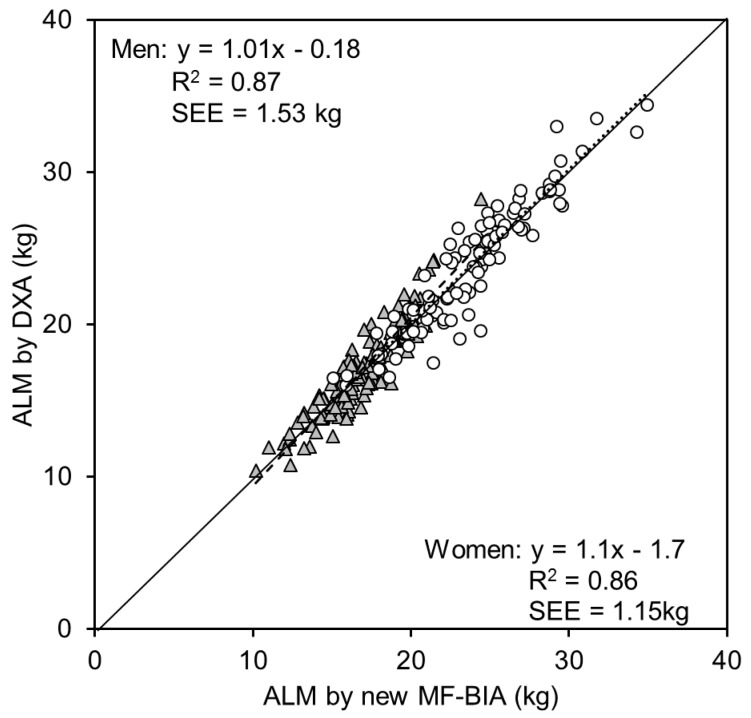
Relationship between ALM estimated by new equation of MF-BIA established with the developing group (222 men and 301 women) against ALM by DXA in validation group with 97 men and 136 women. The determinant coefficients of linear regression analysis were R^2^ = 0.87 and 0.85, respectively. The intercept was not significantly different from zero and the slope was not significantly different from one.

**Figure 6:**
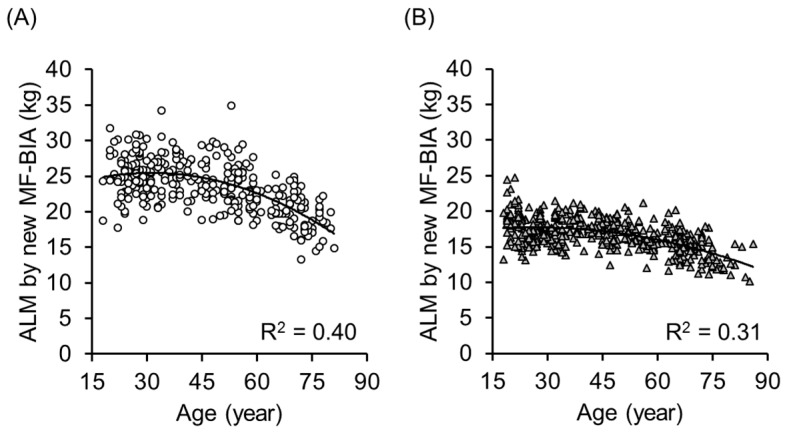
Relationship between age and ALM obtained by new MF-BIA in 319 men (**A**) and 437 women (**B**) aged 18 to 86 years old. The determinant coefficients of quadratic regression analysis were R^2^ = 0.40 and 0.31, respectively.

We applied the equations to the cross validation group (97 men and 136 women). Figure 5 shows the association between ALM estimated by the new equation using MF-BIA and ALM measured by DXA in the cross validation group. The determinant coefficients of linear regression analysis were R^2^ = 0.87 and 0.86, respectively. The SEE was 1.53 and 1.15 kg in men and women, respectively. The intercept was not significantly different from zero and the slope was not significantly different from one (*p* < 0.05). There were no significant differences between measured and estimated ALM both in men (*p* = 0.57) and women (*p* = 0.24) by the paired *t*-test. We calculated the skeletal muscle index (SMI) as follows: SMI = ALM/Ht^2^, and the determinant coefficients of the association between SMI by DXA and SMI by MF-BIA in the validation group were R^2^ = 0.69 and 0.67, in men and women, respectively. Figure 6 shows the correlation between age and ALM obtained by the new MF-BIA and the correlations with age were very similar to that of DXA (Figure 1 and Figure 6).

Furthermore, we measured MF-BIA of 1624 men (mean ± SD; height 171.5 ± 5.8 cm, weight 69.8 ± 12.8 kg, BMI 23.7 ± 4.0) and 1368 women (158.3 ± 5.3 cm, 54.4 ± 9.5 kg, BMI 21.7 ± 3.7) aged 18 to 40 years to establish sarcopenia cutoff values for the Japanese population. The estimated ALM was 25.1 ± 3.3 and 17.0 ± 1.9 kg in men and women, respectively. The SMI were 8.5 ± 0.8 and 6.8 ± 0.5 kg/m^2^, and thus the sarcopenia cutoff values (mean minus 2SD) were 6.8 and 5.7 kg/m^2^ in men and women, respectively.

## 4. Discussion

The aims of the preset study were: (1) to develop and validate an equation to estimate ALM using a standing-posture 8-electrode MF-BIA using theoretical age-independent models, and (2) to establish sarcopenia cutoff values (mean minus 2SD) using a large scale database of Japanese persons aged 18 to 40 years. We developed and validated an age-independent theoretical MF-BIA equation while totally disclosing the theoretical background and development process. The accuracy and precision of the established equation seems reasonable in spite of only using the impedance indices in the regression model.

There are many reports in the literature validating consumer or professional models of BIAs (single-frequency, multi-frequency, whole body or segmental measurements) [43,44]. However, approximately one-half of the papers examined the validity of the output value from BIA software in which the equations have not been disclosed. Thus, it is very difficult to discuss the etiology of the differences between BIA devices or software programs and/or the reasons for the validation results.

One previous study developed an equation and validated it for TANITA BIA in Japanese older adults aged 65 years and over. The equation was simple and used only 50 kHz data, as follows: ALM = (0.197 × (Ht^2^/Z_50_)) + (0.179 × Weight) − 0.019 for men, and ALM = (0.221 × (Ht^2^/Z_50_)) + (0.117 × Weight) + 0.881 for women [38]. The impedance index (Ht^2^/Z_50_) has been used for estimating ALM and/or SMM in many articles. However, when we applied this equation to individuals over a wide age range (18 to 86 years), the equation underestimated ALM significantly in the younger population. We examined the mechanism of this underestimation. The Ht^2^/Z_50_ did not seem to reflect age-related ALM decline, because Ht^2^/Z_50_ was not correlated with age (R^2^ = 0.11 and 0.04, in men and women, respectively) compared with DXA (R^2^ = 0.42 and 0.29, in men and women, respectively), which led to age-related underestimation of the BIA.

To solve the problem, we introduced other variables-the impedance ratio of Z_250_ against Z_5_ (Z_250_/Z_5_), an index of ECW/TBW. Previously, Yamada et al. [32,34,39,40,41] indicated that the impedance ratio of low to high frequency strongly correlated with age and muscle strength, and suggested that the impedance ratio is an important marker of muscle quality. They stated that the impedance ratio reflects the ratio of actual muscle cell mass against total body water. In the present study, Z_250_/Z_5_ correlated significantly with age (R^2^ = 0.37 and 0.23 in men and women, respectively). In the multiple regression model 2, Z_250_/Z_5_ significantly improved the accuracy of ALM prediction, which means that it is essential to take into account the water distribution within the body to estimate ALM by BIA.

In addition, it is well known that BIA is affected by edema or inter-segmental water shift [35,36,37,45], particularly in peripheral segments, such as the ankle and wrist. If a person has edema, in particular peripheral edema, the BIA overestimates actual ALM. To solve this problem, previous studies have used proximal electrode placement locations, such as the knee and elbow. The proximal methods were effective for accurate estimation of body composition, but a major problem is that the proximal methods require additional adhesive-type electrodes on the knees and elbows. This takes time and increases costs, and requires experts who are familiar with anatomical landmarks. We, therefore, tried to solve the problem without using proximal electrode measurement methods. We hypothesized that the index of 1/Z_5_ or 1/Z_50_ may be applied as an adjusting variable in this situation. In the multiple regression model 3, 1/Z_50_ significantly improved the accuracy of ALM prediction. This final model did not have multicollinearity (VIF < 2.5) and had high determinant coefficients. The coefficients of 1/Z_50_ were negative, which means the variables work as a suppressor when an individual had possible edema or water shift into peripheral extra-water segments.

We validated the newly developed equation with the cross validation group, which was an independent group from the equation developing group. As Figure 4 shows, the determinant coefficients of linear regression analysis were very high with reasonable SEE values. The regression line was not significantly different from Y = X. The results indicate that the newly developed equation was cross validated.

The second aim of the current study was to establish a sarcopenia cutoff value using the new equation for the Japanese population. For this purpose, we measured MF-BIA of 1624 men and 1368 women aged 18 to 40 years. The estimated ALM was 25.1 ± 3.3 and 17.0 ± 1.9 kg in men and women, respectively. The SMI values were 8.5 ± 0.8 and 6.8 ± 0.5 kg/m^2^, and thus the sarcopenia cutoff values (mean minus 2SD) based on Baumgartener et al. [42] were 6.8 and 5.7 kg/m^2^ in men and women, respectively. For the Japanese population, Sanada et al. [46] established a cut off using DXA (QDR-4500A, Hologic, MA, USA) as 6.87 and 5.46 kg/m^2^ in men and women, respectively. Tanimoto et al. [47] established a cut off using BIA (TANITA MC-190) of 7.0 and 5.8 kg/m^2^ in men and women, respectively. Yoshida et al. [38] used a BIA (MC-190) and suggested cut offs of 7.09 and 5.91 kg/m^2^ in men and women, respectively. Yamada et al. [48] established a cut off using a BIA (Inbody 720, Biospace) and reported values of 6.75 and 5.07 kg/m^2^ in men and women, respectively. It is important to note that Yamada et al. [49] reported that if we used the manufacturer’s own formula, which is undisclosed, different MF-BIA devices provided different SMI values, but if we used one disclosed equation, even with different devices, MF-BIAs provided the same SMI values over a wide age range (18 to 89 years). The sarcopenia cut off value established in the current study was very similar to the value from the previous publication using DXA [46], and within the range previously published in the literature. Thus, we consider that the values of 6.8 and 5.7 kg/m^2^ from 1624 men and 1368 women, aged 18 to 40 years, can be reasonably used as sarcopenia cut off levels for the Japanese population.

Some limitations of the current study should be noted. First, in previous literature reports, many researchers established and disclosed various equations using professional BIA devices for estimating body composition [17,43]. We did not examine these because there are too many published equations to examine them all, the majority of them are not designed for the Asian population, at least half of the equations include age, and these equations were developed against multiple different gold standards (MRI, CT, underwater weighting, stable isotope dilution, and DXA) for estimating different body composition parts. We cannot state the superiority of the current equation compared with previously established and disclosed equations, which was not the focus of the current analysis. Second, EWGSOP stated that CT and MRI are gold standards for estimating SMM, and ALM of DXA is considered to be an alternative preferred method [7]. However, whole body CT scan requires a high level of radiation exposure, and whole body MRI requires time-consuming post-scan image processing to obtain SMM; thus, we used ALM derived from DXA as the reference. Theoretically, ALM of DXA reflects whole lean tissue mass and is not perfectly equal to SMM. In addition, muscle quality and muscle composition change with aging [50,51,52,53,54,55,56,57], but the current study does not examine these effect. As another limitation, Prado et al. [58] stated that a single cutoff may not suitable for all ages; they used a large dataset of DXA ALM with 13,236 individuals from the 1999 to 2004 NHANES cohort and LMS curve-fitting procedure to establish age and gender specific cutoffs. Our current sample size is much lower than the previous study, thus we need collect more data to develop age and gender specific cut offs based on the LMS method. Further research is needed. Strengths of this study include the adequate sample size to develop and validate a new equation and establish sarcopenia cut off values, and the theoretical equation development in the present study.

## 5. Conclusions

In conclusion, we established an age-independent MF-BIA equation for the Japanese population as follows: ALM = (0.6947 × (Ht^2^/Z_50_)) + (−55.24 × (Z_250_/Z_5_)) + (−10940 × (1/Z_50_)) + 51.33 for men, and ALM = (0.6144 × (Ht^2^/Z_50_)) + (−36.61 × (Z_250_/Z_5_)) + (−9332 × (1/Z_50_)) + 37.91 for women. In the cross validation group, the equation had high determinant coefficients (R^2^ = 0.87 and 0.86, respectively) with reasonable SEE (1.53 and 1.15 kg in men and women, respectively). The current study established and validated this theoretical and age-independent equation of MF-BIA for estimating ALM and provided a sarcopenia cutoff value using the equation for the Japanese population. This MF-BIA device is distributed worldwide, thus it is an interesting challenge to develop equations and establish the sarcopenia cut off for various countries with different population as an area of future research. Readers should examine our results and evaluate how they can be interpreted from the perspective of previous studies and the working hypotheses. The current findings and their implications should be discussed in the broadest context possible. Areas for future research may also be identified.

## Figures and Tables

**Table 1:** Physical characteristics and BIA variables of participants for equation developing and validation.

	**Equation Developing Group (222 Men)**			**Validation Group (97 Men)**		
	**Mean ± SD**	**MAX**	**MIN**	**Mean ± SD**	**MAX**	**MIN**
Age	46 ± 17	81	18	49 ± 18	78	18
Ht	167.5 ± 6.8	181.9	147.1	167.0 ± 7.6	184.6	150.0
Wt	64.8 ± 9.5	93.3	45.4	67.0 ± 10.4	99.0	47.3
BMI	23.1 ± 2.8	31.5	16.2	24.0 ± 3.1	31.1	17.6
ALM_DXA_	23.5 ± 3.8	32.3	14.8	23.8 ± 4.2	34.5	16.0
Z_5_	655.2 ± 64.8	837.2	498.7	636.4 ± 62.5	811.1	499.4
Z_50_	573.6 ± 58.0	735.6	437.2	557.0 ± 56.5	719.7	442.1
Z_250_	508.6 ± 52.6	657.2	388.6	494.5 ± 50.3	641.4	391.3
Ht^2^/Z_50_	49.5 ± 5.8	66.9	33.1	50.6 ± 6.5	72.4	34.5
Z_250_/Z_5_	0.776 ± 0.020	0.838	0.724	0.777 ± 0.020	0.830	0.736
1/Z_50_	0.00176 ± 0.00018	0.00229	0.00136	0.00181 ± 0.00018	0.00226	0.00139
	**Equation Developing Group (301 Women)**			**Validation Group (136 Women)**		
	**Mean ± SD**	**MAX**	**MIN**	**Mean ± SD**	**MAX**	**MIN**
Age	47 ± 18	86	18	44 ± 18	85	18
Ht	154.7 ± 6.7	172.4	137.3	155.2 ± 7.1	170.2	138.3
Wt	54.0 ± 7.3	83.6	38.3	54.1 ± 8.3	80.4	32.5
BMI	22.6 ± 3.1	33.7	16.6	22.4 ± 3.0	32.9	16.0
ALM_DXA_	16.5 ± 2.5	24.2	10.8	17.0 ± 3.1	28.2	10.4
Z_5_	730.8 ± 81.2	965.4	536.9	736.7 ± 79.9	995.7	508.5
Z_50_	657.9 ± 73.0	885.1	484.3	661.1 ± 72.6	901.1	464.6
Z_250_	591.0 ± 65.7	799.5	438.7	592.7 ± 66.0	815.0	420.1
Ht^2^/Z_50_	36.8 ± 4.2	49.8	27.1	36.9 ± 5.2	52.1	24.3
Z_250_/Z_5_	0.809 ± 0.018	0.861	0.746	0.805 ± 0.020	0.851	0.742
1/Z_50_	0.00154 ± 0.00017	0.00206	0.00113	0.00153 ± 0.00017	0.00215	0.00111

Ht, height; Wt, weight; BMI, body mass index; ALM_DXA_, appendicular lean mass by dual X-ray absorptiometry; Z_5_, Z_50_, and Z_250_, whole body impedance of 5, 50, and 250 kHz.

**Table 2:** Multivariate analysis: Linear model with ALM as a dependent variable.

**Men (*n* = 222)**		**Coefficients**		**Sig.**	**Collinearity VIF**
		**Unstandardized**	**Standardized**		
		**B**	**Beta**		
1	Ht^2^/Z_50_	0.5153	0.797	<0.001	
	(Constant)	−1.941		0.1401	
	R^2^ = 0.635, SEE = 2.27 kg				
2	Ht^2^/Z_50_	0.4396	0.680	<0.001	1.138
	Z_250_/Z_5_	−62.84	−0.337	<0.001	1.138
	(Constant)	50.58		<0.001	
	R^2^ = 0.735, SEE = 1.94 kg				
3	Ht^2^/Z_50_	0.6947	1.075	<0.001	2.473
	Z_250_/Z_5_	−55.24	−0.296	<0.001	1.152
	1/Z_50_	−10941	−0.513	<0.001	2.254
	(Constant)	51.33		<0.001	
	R^2^ = 0.851, SEE = 1.46 kg				
**Women (*n* = 301)**		**B**	**Beta**		**VIF**
1	Ht^2^/Z_50_	0.3797	0.644	<0.001	
	(Constant)	2.567		0.008	
	R^2^ = 0.415, SEE = 1.88 kg				
2	Ht^2^/Z_50_	0.3346	0.568	<0.001	1.043
	Z_250_/Z_5_	−50.68	−0.378	<0.001	1.043
	(Constant)	45.22		<0.001	
	R^2^ = 0.552, SEE = 1.65 kg				
3	Ht^2^/Z_50_	0.6144	1.042	<0.001	2.14
	Z_250_/Z_5_	−36.61	−0.273	<0.001	1.096
	1/Z_50_	−9332	−0.649	<0.001	2.053
	(Constant)	37.91		<0.001	
	R^2^ = 0.757, SEE = 1.22 kg				
